# How Older Persons and Health Care Professionals Co-designed a Medication Plan Prototype Remotely to Promote Patient Safety: Case Study

**DOI:** 10.2196/41950

**Published:** 2023-04-07

**Authors:** Malin Holmqvist, Axel Ros, Bertil Lindenfalk, Johan Thor, Linda Johansson

**Affiliations:** 1 Department of Public Health and Healthcare, Region Jönköping County Jönköping Sweden; 2 School of Health and Welfare, Jönköping University Jönköping Sweden; 3 Futurum, Region Jönköping County Jönköping Sweden; 4 Jönköping Academy for Improvement of Health and Welfare, School of Health and Welfare, Jönköping University Jönköping Sweden; 5 Institute of Gerontology, School of Health and Welfare, Jönköping University Jönköping Sweden

**Keywords:** co-design, remote, older people, medications, medication plan, patient safety, patient experience, participatory, engagement

## Abstract

**Background:**

Harm from medications is a major patient safety challenge. Most adverse drug events arise when a medication is prescribed or reevaluated. Therefore, interventions in this area may improve patient safety. A medication plan, that is, a plan for continued treatment with medications, may support patient safety. Participation of patients in the design of health care products or services may improve patient safety. Co-design, as in the Double Diamond framework from the Design Council, England, can emphasize patient involvement. As the COVID-19 pandemic brought restrictions to face-to-face co-design approaches, interest in remote approaches increased. However, it is uncertain how best to perform remote co-design. Therefore, we explored a remote approach, which brought together older persons and health care professionals to co-design a medication plan prototype in the electronic health record, aiming to support patient safety.

**Objective:**

This study aimed to describe how remote co-design was applied to create a medication plan prototype and to explore participants’ experiences with this approach.

**Methods:**

Within a case study design, we explored the experiences of a remote co-design initiative with 14 participants in a regional health care system in southern Sweden. Using descriptive statistics, quantitative data from questionnaires and web-based workshop timestamps were analyzed. A thematic analysis of the qualitative data gathered from workshops, interviews, and free-text responses to the survey questions was performed. Qualitative and quantitative data were compared side by side in the discussion.

**Results:**

The analysis of the questionnaires revealed that the participants rated the experiences of the co-design initiative very high. In addition, the balance between how much involved persons expressed their wishes and were listened to was considered very good. Marked timestamps from audio recordings showed that the workshops proceeded according to the plan. The thematic analysis yielded the following main themes: *Everyone’s perspective matters*, *Learning by sharing*, and *Mastering a digital space*. The themes encompassed what helped to establish a permissive environment that allowed the participants to be involved and share viewpoints. There was a dynamic process of learning and understanding, realizing that despite different backgrounds, there was consensus about the requirements for a medication plan. The remote co-design process seemed appealing, by balancing opportunities and challenges and building an inviting, creative, and tolerant environment.

**Conclusions:**

Participants experienced that the remote co-design initiative was inclusive of their perspectives and facilitated learning by sharing experiences. The Double Diamond framework was applicable in a digital context and supported the co-design process of the medication plan prototype. Remote co-design is still novel, but with attentiveness to power relations between all involved, this approach may increase opportunities for older persons and health care professionals to collaboratively design products or services that can improve patient safety.

## Introduction

Harm caused by medications, often known as adverse drug events (ADEs) [1], is recognized by the World Health Organization as 1 of the 3 greatest patient safety challenges [2]. The older population, defined here as those aged ≥75 years, tends to be more prone to ADEs due to a higher prevalence of illness and multiple medications [3]. Errors related to ADEs tend to arise when medications are prescribed or reevaluated [4]. Therefore, interventions in these areas may improve safety. Safe clinical management of medications in older persons may be supported by a plan for continued treatment [5]. Such a plan, a medication plan, articulating what to observe when using medications (expected and adverse effects), when to act, and who should take the initiative when needed, may facilitate resilient performance, that is where a system adjusts to maintain a desired level of performance [6].

Having patients participate in the design of health care processes can improve patient safety [7]. In Sweden, as in many other countries, regulations and national policies strengthen and clarify patients’ position and promote their integrity and participation in health care [8]. Furthermore, persons invited to participate in initiatives aimed at improving patient safety are generally willing to do so [9]. There is still a lack of methods for patients to be involved in efforts to improve patient safety [10]. O’Hara and Lawton [11] outline opportunities to improve patient involvement in design and improvement initiatives for patient safety, highlighting the need to explore ways to engage susceptible patients, such as older persons, on their terms, as they tend to be at the highest risk for adverse events. If they are not involved, it may limit the generalizability of improvement efforts, as they may not reflect the reality of older people. So far, the dominant approaches to involving patients in patient safety initiatives include making them more aware of risks and comfortable with giving feedback about safety concerns rather than having them participate in improvement initiatives [12].

**Figure 1:**
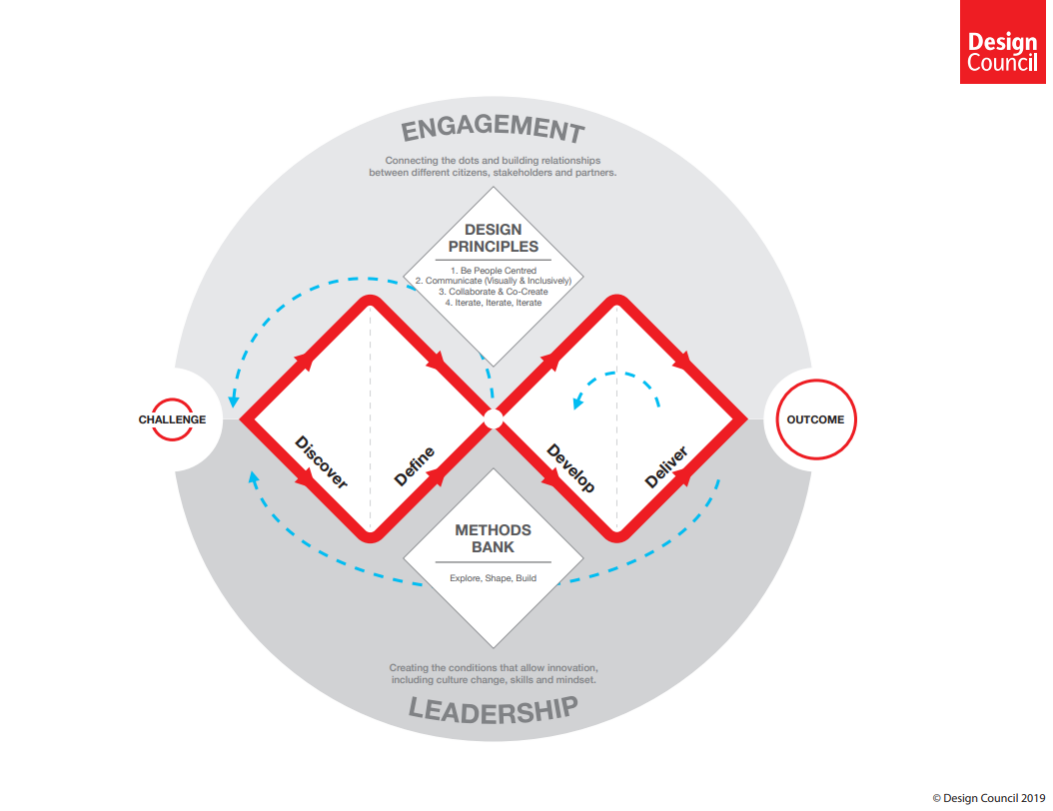
Design Council Double Diamond, created in 2004 (reproduced from Ball et al [21], with permission from The Design Council).

Co-design is an approach that emphasizes patient involvement in the improvement of health care services [13]. It enables participants to share experiences, where each participant’s experience is considered their expertise [14]. A co-design process is powerful, yet challenging, as both patients and health care professionals need to negotiate their roles and balance power between them [15]. Co-design is now an established approach in health care [16]. Even so, initiatives involving older persons are scarce, but some initiatives have addressed the development of different electronic health care tools [17,18] or improvement of specific health care services [19]. There are different frameworks for co-design in health care [16,20], including the Double Diamond from the Design Council, England [21]. The Double Diamond has 4 phases: Discover, Define, Develop, and Deliver (Figure 1). The Discover phase explores the problem space from a service user perspective, whereas the Define phase synthesizes insights to understand how service users are affected by the problem. The Develop phase encourages the exploration of potential solutions to the problem, which in the subsequent Deliver phase are tested and improved before they are incorporated into daily practice.

Although co-design approaches are increasingly common in health care, they are criticized for a lack of evaluation of their effectiveness and cost-effectiveness [22] and are seen as challenging to implement in a busy health care environment. Therefore, the application of any co-design framework must be adapted to the contextual needs of the health care system. The COVID-19 pandemic brought restrictions to co-design approaches, which are usually performed face-to-face with users. Remote co-design may have both limitations and strengths, but it seems possible to perform with similar outcomes and quality as face-to-face co-design efforts [23,24]. Remote co-design initiatives including older persons and health care professionals to support patient safety seem rare, and their potential and limitations are not fully understood. Therefore, we report the experiences of such an initiative concerning patient safety. This study aimed to describe how remote co-design was applied to create a medication plan prototype and to explore participants’ experiences with this approach.

## Methods

### Study Design

We report on the co-design initiative using a case study design [25]. Case study methods are useful when exploring efforts to improve patient safety in complex systems such as health care [26]. Qualitative and quantitative data were first analyzed separately and then included in a side-by-side comparison in the discussion.

### Participants and Setting

The co-design initiative was part of a large national initiative introducing Patient Contracts, aiming to strengthen patients’ role in health care [27]. A Patient Contract is an agreement, documented in the electronic health record, intended to strengthen the relationship between a patient and caregivers by promoting care coordination, accessibility, and predictability. To achieve a variety of perspectives and experiences [28], we sought a balanced group composition with an equal number of older persons (aged >75 years), next of kin, general practitioners, and nurses working in municipality-based home health care. Participants were recruited through existing contacts within the initiative Patient Contract in 1 regional public health care system in southern Sweden, serving a population of 350,000 residents [27]. Inclusion required availability to participate in all 3 parts of the initiative, adequate communication capability in Swedish, and access to and comfort in using the internet. There were no explicit exclusion criteria for this study. Gender, age, and number of medications were noted for older persons; for health care professionals, gender and years in the profession were noted.

The initiative included 14 participants (Table 1): 3 general practitioners who had worked for 5-39 years as physicians, 6 nurses who had worked for 4-35 years as registered nurses, and 5 older persons aged 72-82 years and using 3-8 medications daily. One of the older persons also reported having the experience of being next of kin to a person taking medications.

**Table 1:** Number of participants and data collected at each step of the study (N=14).

Session	Participants, n (%)				Data collected	
	Older persons, n (%)	General practitioners, n (%)	Nurses, n (%)			
Workshop 1 (n=14)	5 (36)	3 (21)	6 (43)	Audio recording with timestamps, Zoom Polls		
Workshop 2 (n=14)	5 (36)	3 (21)	6 (43)	Audio recording with timestamps, Zoom Polls		
Survey (n=13)	5 (38)	3 (23)	5 (38)	Digital questions, closed and in free-text form		
Interviews (n=7)	4 (57)	1 (14)	2 (29)	Audio recordings		

### The Co-design Initiative

**Figure 2:**
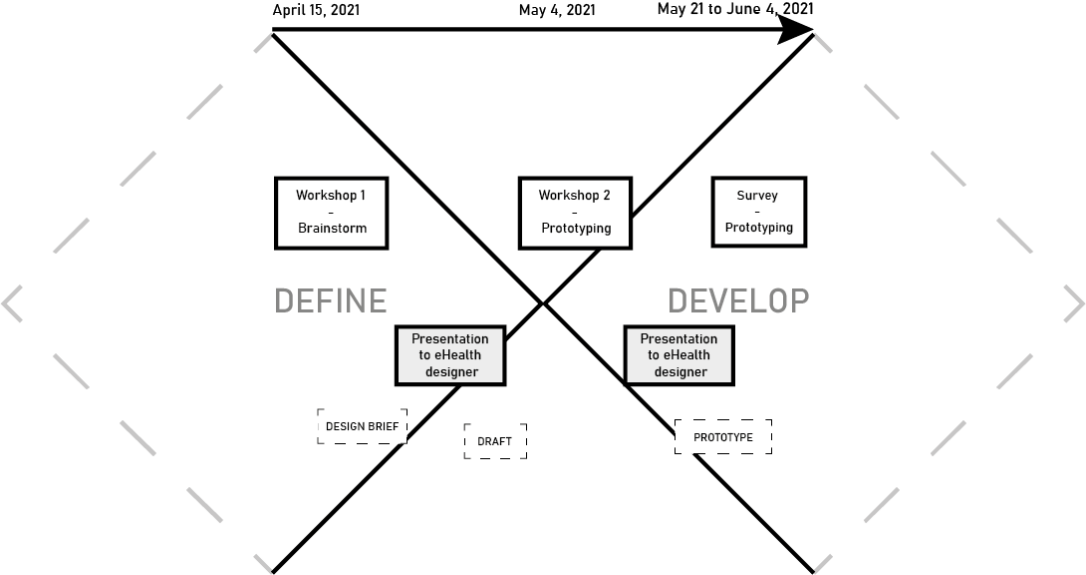
The structure of the co-design initiative.

The co-design initiative (Figure 2) aimed to create a prototype, that is, a model of a proposed solution, for a medication plan incorporated in the electronic health record to support older persons and health care professionals jointly in using and monitoring medications. This initiative focused on the Define and Develop phases in the Double Diamond framework and was informed by the guide “Design methods for developing services” by the Design Council [29]. The first phase of the Double Diamond, Discover, has been performed before [30,31], and the last phase, Deliver, will be performed later. This co-design initiative was performed over a 2-month period and included 3 sessions: 2 workshops via the web-supported Zoom (Zoom Video Communications) videoconferencing software and 1 survey session (Multimedia Appendix 1).

Two facilitators, the first author and one quality improvement adviser, guided the participants through the co-design initiative. Two additional persons provided technical support, collected notes on the Padlet Web platform (a digital notice board), recorded audio, and encouraged collaboration during the workshops.

The workshops lasted for 2 hours each and were guided by a minute-by-minute timetable. The timetable was set up by the first and third author (with experience in co-design), together with the quality improvement adviser, detailing all planned activities (Multimedia Appendix 2). The setup for the workshops was pilot-tested to identify and resolve challenges to the digital collaboration. One week before each session, all participants received general information about the session along with specific input: before workshop 1, insights generated in the Discover phase and before workshop 2, the design brief and medication plan drafts and along with the survey, the medication plan prototype. To ensure that everyone received the information, it was sent out by both postal mail and email. Between the 3 sessions, facilitators presented data from the co-design initiative to eHealth designers in the regional public health care system where the initiative was performed, which informed their prototype design.

In the Define phase, including the first workshop, participants were invited to analyze the findings from the Discover phase, when insights about the evaluation of medications were gathered from older persons, nurses, and general practitioners [30,31] along with information from research studies and regulations related to the initiative. Furthermore, they were asked to synthesize their analysis into a number of opportunities for a medication plan. Brainstorming was used to gather ideas and build a shared understanding of the orientation of the group. After the session, the first author formed a design brief, that is, a core reference point based on gathered data produced during the session, and presented it to the eHealth designers, who used it to assist in preparing medication plan drafts, that is, preliminary prototypes.

In the Develop phase, including the second workshop and the concluding survey, participants were invited to develop the drafts further into 1 prototype by designing components in detail and iteratively refining the draft. Experience Prototyping, a way to test and refine a solution in feedback loops with potential users, with made-up fictitious patient cases, was used to enable participants to gain first-hand understanding of the drafts and gather feedback from them. After the second workshop, the first author gathered data produced during the session and presented it to the eHealth designers, further informing the design of the medication plan prototype. This prototype was sent out to all participants together with a survey, open for 2 weeks, to collect final feedback on the prototype.

### Data Collection

This case study of the co-design initiative draws on quantitative and qualitative data (Table 1).

Audio recordings of the workshops were transcribed verbatim and marked with timestamps. Zoom Polls (ie, questions) about the participants’ experience of the workshop, with fixed response options on a 4-grade Likert scale, ended each workshop. A survey, created in the web-based survey tool esMaker NX3, was sent out to all participants to collect feedback and reflections related to the co-design activities, participation in the activities, and practicalities of participating. It consisted of 7 questions with answers on a 10-grade Likert scale and with a possibility to add free-text comments, 1 yes or no question, and 7 additional free-text questions. The participants had 2 weeks to respond and received 2 reminders, after 1 week and on the last day for completion. All participants were invited to participate in an individual semistructured interview on Zoom. The interview guide (Multimedia Appendix 3), developed by the research team based on the findings from the survey, included questions about the prototype and the co-design process. The interviews were audio recorded, transcribed verbatim, and lasted between 21 and 46 minutes (30:37 SD 9:13).

### Data Analysis

Quantitative data from the Zoom Polls and survey were analyzed using descriptive statistics. Marked timestamps in the workshop audio recordings were compared with the original timetable.

Qualitative data from the free-text responses in the survey and the transcribed audio recordings from workshops and interviews were analyzed together through thematic analysis, as outlined by Braun and Clark [32,33]. An inductive approach was applied to look for patterns, that is, to identify themes addressing the underlying meanings of data. To support the analysis, transcriptions and free-text responses were gathered using the NVivo software (QSR International). Initially, the material was read and reread to strengthen familiarity with data. The first author generated the initial codes and gathered them into potential themes. Then, the first and last authors reviewed the initial codes and themes and presented them to the entire research group to define the final themes. The entire research team contributed to writing and rewriting the descriptions of the themes and to generating the report.

### Ethics Approval

This study was approved by the Swedish Ethical Review Authority (dnr 2020-04781) and adhered to the Declaration of Helsinki [34]. All participants received written information regarding the aim and arrangement of the study and provided written consent before the first session. Data were deidentified to maintain confidentiality and were presented so that no individual could be identified. Data from the study were kept secure at Region Jönköping County.

## Results

### Quantitative Data Regarding Experiences of the Remote Co-design Initiative

Using Zoom Polls, participants (13/14, 93%) assessed the first workshop as good (9/13, 69%) or very good (4/13, 31%). The participants’ (12/14, 86%) experience after the second workshop was fairly good (2/12, 17%), good (5/12, 42%), or very good (5/12, 42%). For the yes or no question in the survey, they (11/12, 92%) responded that the initiative corresponded to the aim, that is, to develop a medication plan prototype together; 1 respondent did not know. On the following survey questions, participants (13/14, 93%) responded with answers on a 10-grade Likert scale (Table 2). They reported being able to speak to the extent they wanted (median response 9) and that the views they expressed were considered when developing the prototype (median response 9). On a scale of 1 being very bad and 10 being excellent (Table 3), participants’ overall experiences of participating in the co-design initiative, the balance between how much all involved expressed their wishes, and the information provided to facilitate participation were close to excellent (median response 9). On the same scale, the participants indicated that the practical parts of the workshops facilitated participation and that the balance between how much all involved were listened to was almost excellent (median response 8).

Marked timestamps from the workshop audio recordings showed that both workshops ended according to the schedule (Multimedia Appendix 2). Presentations of activities took in general less time than planned in both workshops. The background introduction in the first workshop took longer than planned. In the second workshop, the introduction to and test of the digital notice board took longer than planned. The time for individual reflections was shorter than planned, whereas prioritizing the requirements for the medication plan took more time than planned.

**Table 2:** Answers from the survey (Q=question number) on a 10-grade Likert scale (13/14, 93%)^a^.

Item	1	2	3	4	5	6	7	8	9	10	Do not know	Median
Q4. In the workshops I was allowed to speak to the extent that I wanted	0	0	0	0	0	0	1	3	4	5	0	9
Q2. The views I expressed in the workshops were taken into account in developing the medication plan prototype	0	0	0	0	0	0	1	3	4	3	2	9

^a^1=do not agree to 10=totally agree.

**Table 3:** Answers from the survey (Q=question number) on a 10-grade Likert scale (13/14, 93%)^a^.

Item	1	2	3	4	5	6	7	8	9	10	Do not know	Median
Q1. What is your overall experience of participating in the work of creating a medication plan prototype?	0	0	0	0	1	0	1	4	5	2	0	9
Q6. How did you experience the balance between how much older persons, general practitioners and nurses expressed their wishes?	0	0	0	0	1	2	0	3	3	4	0	9
Q7. How did you experience the balance between how much the views of older persons, general practitioners and nurses were listened to?	0	0	0	0	1	0	2	3	4	1	2	8
Q8. How did you perceive that information provided before, during, and after the workshops facilitated your participation?	0	0	1	0	0	0	2	3	5	2	0	9
Q9. How did you experience that the practical parts of the workshops (that is, use of the digital platform, the facilitators’ actions) facilitated your participation?	0	0	0	0	1	2	1	3	4	2	0	8

^a^From 1=very bad to 10=excellent.

### Qualitative Data Regarding Experiences of the Digital Co-design Initiative

Thematic analysis included interviews, workshops, and free-text responses to questions in the survey. A total of 3 main themes and 11 associated subthemes were identified, which described the experiences of the approach (Textbox 1). The main themes *Everyone’s perspective matters*, *Learning by sharing*, and *Mastering a digital space* encompass conditions of importance for establishing a permissive, dynamic, and appealing remote co-design process, as described in the following text.

*Everyone’s perspective matters* reflects the experiences of a permissive environment during the co-design process, which allowed the participants, especially older persons, to be involved and share viewpoints that were taken into account when developing the prototype.

On the basis of their different perspectives and backgrounds, the participants experienced that they were *contributing from multiple viewpoints* to the design of the prototype. The purpose, that is, to improve medication safety, seemed to be important when participants decided to participate, as it was something worthwhile for them to affect. Contributing with different viewpoints in the workshops was considered to add broader input to the prototype, something not possible to extract from the existing literature. With different perspectives, both as individuals and as representatives of a group of people, the participants complemented each other, which means that the prototype was created collaboratively:

I think that…what I heard from the nurses in other municipalities as well, it is pretty much the same thoughts as we shared. But I think we complemented each other quite well.

Interview—nurse

The atmosphere in the workshops was *inviting to dialog*. Together, facilitators and participants created opportunities for the exchange of perspectives by inviting all participants, regardless of who, to provide input and by allowing everyone to speak:

Yes, but I think we had the opportunity to speak, especially at the group meetings.

Survey—older person

Participants’ viewpoints were taken into consideration in the development of the prototype, meaning that everyone counted. Even so, concerns were raised that some people, regardless of their roles, occupied more space than others. At the same time, different ways to inform the development of the prototype during the process, that is, by dialog, Zoom Polls, and note boards were appreciated as they gave participants opportunities to provide input into the process, including ideas that popped up between sessions

**Themes and subthemes.**

Everyone’s perspective matters

- Contributing from multiple viewpoints
- Inviting to dialog
- The voice of medication users

Learning by sharing

- Acknowledging each other’s daily life
- Creating shared understanding
- Reaching coherence

Mastering a digital space

- Meeting remotely is the future
- Building relationships remotely
- Structuring the work
- Digital hassles
- Allocating time

*The voice of medication users* stood out and was considered to be of extra importance, meaning that services in health care must be adjusted to make sense for both patients and health care professionals. Some health care professionals even highlighted that older persons should have had even more impact than they had in the co-design initiative. In contrast, the older persons thought that health care staff’s requirements must outweigh, as medication plans mainly concerned their way of working in daily practice. They also stated that they as patients had a responsibility to contribute to improving health care:

And I think so…patient’s own responsibility. I would like to mention that. You cannot be passive in healthcare. Because then you are gone very quickly. You have to be…uh, at the forefront, updated, inquisitive. Ask uncomfortable questions. And you want an answer.

Interview—older person

Although it was emphasized by the health care professionals that the older persons in this initiative were particularly knowledgeable, that is, “expert patients,” there were also concerns that the balance between patients and health care professionals might be to the disadvantage of the patients. Older persons might be frail, and medical terms and jargon in discussions can make it difficult for them to be completely involved.

*Learning by sharing * highlights the dynamic process of learning and understanding by sharing daily life experiences and realizing that despite different backgrounds, participants could reach consensus about the requirements for a medication plan.

Gaining knowledge about how things work out in different contexts and to *acknowledge each other’s daily life* was experienced as fruitful, meaning that the participants may not normally be aware of the situations of other patients or health care professionals:

You get an insight into each other’s practice and can jointly reach something that works for everyone.

Survey—nurse

The fictitious cases used in the workshops, whether the participants considered them familiar or not, worked out well for participants to reflect upon. The sharing of each other’s everyday practices contributed to individual learning and showed how other participants looked at the difficulties associated with medications. This learning provided insight into the desirable requirements for the medication plan and also into the challenges to come, such as medication prescribers having enough time when introducing a new way of working together.

Having a 2-way dialog with the possibility of providing direct responses to one another helped in *creating shared understanding.* The participants saw creating something together as valuable and good, meaning that it supported the development of the prototype:

Yes, it [cocreation] is that people with different experiences, different lengths of experience, get the opportunity to work together and learn from each other. And build…build something together with…the platforms you have.

Interview—older person

Participating in a dialog appeared to increase interest in the topic itself, generating increased curiosity about medication treatment in general. This approach was also considered worth applying to other improvement initiatives. However, concerns about missing out on the perspectives of groups other than those included, such as homecare staff and pharmacists, were raised, meaning that home health assistants (who were not represented in the co-design initiative) have valuable additional insights into everyday practice close to older persons.

The participants were *reaching coherence* for the final prototype, meaning that they seemed to empathize with each other’s needs to make the prototype applicable:

There was consensus around it. And I think that is also a very good thing about co-creation, that…just that we realize that there is a consensus.

Interview—older person

The prototype corresponded well with participants’ needs and expectations. They stated that it also seemed to live up to the requirements of the group as a whole. Understanding that different visions are not always profession related but person related was another experience the participants seemed to gain, as they observed that there appeared to be no “us and them.” Similarities in how to manage things applied to people, not profession.

*Mastering a digital context* focuses on the balance between opportunities and challenges the digital platform offers, such as building an inviting and creative environment for co-design in a web-based context.

Participants argued that *meeting remotely is the future*, as easy access from home or the workplace leads to efficient meetings:

Yes, many times, you have slightly more pithy comments and you listen a little more to others as well.

Interview—general practitioner

The digital platform made it possible to join without losing valuable working hours traveling. The accessible platform was considered a proper alternative even after meeting restrictions, imposed during the COVID-19 pandemic, were lifted. The digital space provided ample room for reasoning and possibilities to interact in other ways, for instance, via digital chat.

At the same time, *building relationships remotely* seemed to be more difficult, as the context challenges the way we normally build relationships. For example, the participants commented that informal chats during a coffee break, which help people get to know each other, were hard to imitate digitally. Moreover, having all faces side by side on a screen made it difficult to remember who was who and what role the person had (Textbox 2).

Knowing if someone was behind a “black screen”; the interpretation of silence in the digital room; and etiquette, such as knowing when to speak or not, seemed to be more complicated remotely, thereby hampering social interaction.

*Structuring the work*, such as moderating and giving clear instructions before and during each session, was desirable according to the participants and something they reported experiencing. The structure and quality of the workshops seemed to be related to the facilitators’ ability to provide guidance and instructions to the participants, for example, how to mute the microphone, answer Zoom Polls, or get in and out of breakout rooms. At the beginning of the process, some participants experienced that they did not know what was expected of them, which created uncertainty, but the support they received at the start made them feel comfortable and on track.

Technical problems, such as not being able to connect to workshops in time, not being able to present the right screen, or not understanding how to manage the digital room, contributed to *digital hassles* (Textbox 3).

**Transcription from a breakout room in workshop 2 (P=older person and F=facilitator)**

P: Question from X [name]. I wonder, how many of us are in our group?

F: You are four in your group.

P: There are four of us? Yes, that is good. It is a new outfit today, so you never know…

F: Exactly. Y [name] is a nurse. Z [name] is a doctor. And X [name] and W [name] are users of medications.

**Transcription from a breakout room in workshop 1 (P=older person and F=facilitator)**

F: Well I think…he probably managed to press ‘Join’ there. So we will…we will wait…we are waiting for some more to come. It is two more…

P: Yes.

F: We will see if she succeeds with that…Or what happens. But we can start a little bit then…There comes x [name] too. Welcome.

Some participants had more problems than others, and guidance was often required to resolve these situations. This could take extra time and contribute to some participants missing parts of the workshop.

*Allocating time* for cocreation was experienced as essential, meaning that during the workshops time seemed to pass quickly, whereas having time for discussion and reflection was considered important for the outcome. Worries were raised that time was hardly ever adjusted for difficult issues such as those in this initiative. Some participants also argued that more time would have helped older persons to provide input.

## Discussion

### Principal Findings

User involvement in initiatives aiming at supporting patient safety is novel. Therefore, this study describes a remote co-design initiative, including both older persons and health care professionals, and highlights the experiences of the approach. Our findings indicate that remote co-design can be a complement or substitute for face-to-face co-design sessions but requires careful planning and adjustments in action throughout the process to allocate sufficient time for cocreation. Even so, the approach allowed an accessible environment for the participants, and the chosen co-design framework seemed to work well in a digital context. The participants had a positive experience of participating in the co-design initiative and thought it allowed a permissive environment where everyone’s perspectives mattered. Sharing of everyday life created learning and resulted in the participants gaining awareness of possible risks and strategies that could contribute to safer medication treatment.

### Comparison of Data and Comparison With Previous Work

In the quantitative results, participants rated the overall experience of the second workshop lower than the first, and the time for individual reflections in the second workshop was shorter than planned. In addition, the thematic analysis identified that participants considered time for dialog important and that lack of time could be a limitation, especially for older persons. To the best of our knowledge, the optimal duration of a co-design session is unknown. The time frames for these workshops were therefore a prediction, limiting the risk of digital fatigue [23], while allocating enough time for interaction and achieving adequate input on the prototype. The participants in this study experienced that they had the opportunity within the given time frame to hear and respond to others’ input directly. To find time for co-design is a matter of discussion, as co-design generally requires more time compared with top-down approaches, such as individual or group interviews, used to inform the design of a service [20,35]. Using participants’ time efficiently is advantageous, especially because a high workload makes it difficult to engage health care professionals in co-design initiatives [36]. The remote approach made it possible to participate without spending time on travel. Apart from saving time, remote initiatives may broaden participant groups, inviting those who really want to participate instead of only those who have the capacity and time to attend physical meetings [23,24]. Previous studies have shown that remote co-design has pros and cons [23,24], and this study revealed that digital hassles took time and adversely affected the timetable. Even so, managing new technology is not limited to aspects of age [37]. Individual user perspectives are more important to address than chronological age and are worth paying attention to in future remote co-design initiatives.

Even if the time allocated for cocreation might have been short, participants reported that they had opportunities to speak as much as they wanted. The workshop structure, guided by the Double Diamond framework, and the digital context seemed to form a welcoming environment, where facilitators and participants together created a space for the exchange of perspectives and ideas where everyone was empowered to speak and provide input. This mirrors another co-design study from Australia [23], suggesting that the web-based meeting space may break down power imbalances and establish equal participation. Facilitators can balance power between participants and encourage vulnerable members in a group to express their opinions [38]. As in many other co-design activities [39], the facilitators and participants in this initiative had no specific training in co-design. Nevertheless, our findings show that the participants were satisfied with the facilitation both during workshops and in between.

Both health care professionals and older persons in this study emphasized the importance of considering the views of patients when creating new services or products. However, the findings included statements that health care staff’s requirements must be more important than those of patients and also revealed concerns that building relationships remotely can be more challenging than face-to-face meetings. To constantly reflect on power relations in co-design initiatives is therefore essential to empower a balanced relationship between involved persons [15,40]. Future remote co-design initiatives conducted in the context of patient safety need to anticipate this, as an imbalance between patients and health care professionals may threaten the outcome. If health care organizations invite patients to co-design, health care professionals must share power with them and work responsibly to overcome barriers on equal terms [41].

On the basis of the results of this study, we cannot determine whether a remote co-design initiative with older persons supports patient safety. The participants experienced individual learning during the initiative, and by allowing patients, together with health care professionals, to address safety concerns, they might gain higher awareness of risks in their everyday life [42]. Other studies have shown that patients actively involved as cocreators of resilience at the micro, meso, and macro levels develop their own strategies to reduce harm and use their capabilities to contribute to safer care [43,44]. Patient participation in initiatives aimed at improving patient safety is still insufficiently explored, possibly because of challenges that arise when trying to involve patients in the complexity of health care processes [11]. Sharing of everyday practice provided the participants with insights into why special requirements were called for in the medication plan and also revealed possible challenges for future implementation. In approaches aimed at improving health care quality and patient safety, it is important to address organizational human factors and encourage the active and adaptive role of users [45]. The Systems Engineering Initiative for Patient Safety is a human factors systems approach to patient safety, which acknowledges the importance of considering the whole system for an intervention to have a sustainable impact on patient safety [45,46]. The participants in this initiative, representing different parts of the health care system, experienced that they contributed to the development of the prototype from multiple perspectives, such as patients and health care professionals. Their shared understanding, developed through this process, contributed to their common view of what was needed for the prototype to work well for everyone. Therefore, we conclude that co-design initiatives that include everyone’s perspectives and create learning for those involved may, also in a remote approach, inform the development of new products or services to improve patient safety.

### Strengths and Limitations

We aimed to ensure trustworthiness, credibility, confirmability, dependability, and transferability were considered throughout this case study [47]. The case study design, with a thorough description of the initiative and using well-known qualitative and quantitative methods, contributed to *dependability* of the results. To ensure consistency, a pilot test of the setup for the workshops was conducted and evaluated, resulting in some minor adjustments in the timetable. The first author performed all interviews with a semistructured interview guide to promote consistency.

Owing to the broad approach to recruiting participants, it is unknown how many people choose not to participate and why. We aimed for a mix of older persons, next of kin, and health care professionals [28], which was achieved, except that no persons participated solely as next of kin. The participants were recruited within the initiative Patient Contract and may therefore be prone to working together to strengthen the patients’ role in health care. In addition, older persons may be considered extra knowledgeable, and they had previous experience with digital meetings. Persons unable to communicate in Swedish or use a computer were therefore not included in this initiative, and concerns that digital initiatives may exclude some people have been raised before [23]. Consequently, the inclusion criteria used in this initiative need to be considered when interpreting the results, as they may affect *transferability* to other co-design initiatives with older persons.

Objectivity is important when considering the *confirmability* of data. The first author and one quality improvement adviser guided and facilitated the co-design initiative. Both had met some of the participants before in other projects, meaning they were known to each other. To ensure objectivity in the study findings, peer debriefing was used in the thematic analysis, where the first and last authors first reviewed themes together and then presented them to the author group to rewrite the final descriptions. The quality improvement adviser was not involved in the data analysis.

We believe that the multidisciplinary author group, with different experiences in patient safety and co-design, contributed to the study’s *credibility*, as preunderstanding is helpful in case studies and when interpreting if the findings are real and accurate. Linking and comparing our findings with each other and prior research also ensured the accuracy of our findings.

### Future Directions

This remote co-design initiative has resulted in a medication plan prototype. A future study could address the last phase in the Double Diamond framework, the Deliver phase, and evaluate the medication plan prototype according to usability and safety.

Future studies could also expand on the remote co-design approach’s possibilities to broaden the participant group and increase opportunities for older people and health care professionals to meet and collaboratively improve patient safety.

The web-based meeting space seems to support an inviting environment and establish equal participation, which allows participants to address safety concerns and contribute to safer care. Therefore, research to increase knowledge on how patients can be further involved in remote co-design initiatives aimed at improving patient safety would be helpful.

### Conclusions

Participants experienced that the remote co-design initiative was inclusive of their perspectives and facilitated learning by sharing experiences. The Double Diamond framework was applicable in a digital context and supported the co-design process of the medication plan prototype. Remote co-design is still novel, but with attentiveness to power relations between all involved, this approach may increase opportunities for older persons and health care professionals to collaboratively design products or services that can improve patient safety.

## Appendix

Multimedia Appendix 1 Survey.

Multimedia Appendix 2 Timetable with planned activities.

Multimedia Appendix 3 Interview guide.

## Abbreviations

ADE: adverse drug event

## Data Availability

The data generated during this study are available from the corresponding author upon reasonable request.
