# Supplementary material for: How Older Persons and Health Care Professionals Co-designed a Medication Plan Prototype Remotely to Promote Patient Safety: Case Study
Source: JMIR Aging. 2023 Apr 7;6:e41950. doi: 10.2196/41950 (PMC10131987; doi:10.2196/41950)
Supplement: Multimedia Appendix 1 [file aging_v6i1e41950_app1.docx]

**Survey**

**Co-design of a meditation plan**

**Introduction**

As a final part of the co-design initiative, aiming to co-create a medication plan, we ask you to take part of the medication plan prototype, based on the medication list in the electronic health record, and then answer the survey (19 questions in total). One part of the survey addresses the medication plan prototype. The other part addresses values and challenges with the co-design work.

You choose by yourself how much you want to comment on the questions.

The survey is estimated to take 20-30 minutes to complete.

**Overarching question**

What was your "role" in this work?
[Multiple choices: person with medications/ next of kin/ municipality based nurse/ physician]

**Questions related to the prototype**

1. As a group, you have identified that treatment goal as well as when and how the medications will be evaluated are most important content in a medication plan.

1a. Do you agree on that these contents are most important?

[Single choice: Yes/ No/ Do not know + Open comment box]

1b. Do you find these contents in the medication plan prototype?

[Single choice: Yes/ No/ Do not know + Open comment box]

1c. Is there anything in the medication plan prototype you still think is missing? If yes, what?

[Open question]

2. As a group, you have wished for having the medication plan integrated within the medication list.

2a. Based on the medication plan prototype, do you agree on that a medication plan should be integrated in the medication list?

[Single choice: Yes/ No – register where else + Open comment box/ Do not know]

2b. What are the pros and cons with the medication plan prototype?

[Open question]

3a. To what extent do you feel that the prototype meets your objectives for a medication plan?

[Grade 1-10; 1 = not at all, 10 = to a very large extent// Do not know]

3b. What needs to be adjusted for your goals to be met 100%?

[Open question]

4a. Do you think that the time it would take to create/ maintain the medication plan at a healthcare appointment corresponds to the medication plan’s potential contribution to patient safety?

[Grade 1-10; 1 = not at all, 10 = to a very large extent// Do not know]

4b. What needs to be adjusted for you to feel that it contributes to patient safety to 100%?

[Open question]

5a. Would you consider using the prototype as a medication plan?

[Grade 1-10; 1 = not at all, 10 = to a very large extent// Do not know]

5b. What needs to be adjusted for you to feel that it I usable to 100%?

[Open question]

6. Imagine a perfect medication plan; how well does the medication plan prototype match your imagination?

[Grade 1-10; 1 = worst possible match, 10 = best possible // Do not know]

7a. To what extent do you assess that the medication plan prototype may contribute to increased patient safety in medication treatment?

[Grade 1-10; 1 = not at all, 10 = to a very large extent// Do not know]

7b. Describe, with your own words, your thoughts about how the medication plan prototype could affect patient safety. We appreciate both positive and negative feedback.

[Open question]

8a. To what extent do you assess that the medication plan prototype is useful for you?

[Grade 1-10; 1 = not at all, 10 = to a very large extent// Do not know]

8b. Describe, with your own words, your thoughts about the usability of the medication plan prototype. We appreciate both positive and negative feedback.

[Open question]

**Questions related to the co-design work**

1a. What is your overall experience of participating in the work of creating a medication plan prototype?

[Grade 1-10; 1 = a very bad experience, 10 = the best possible experience // Do not know]

1b. Motivate, with your own words, your experience of participating in this initiative. We appreciate both positive and negative feedback.

[Open question]

2. Has the initiative fulfilled its aim, that is to jointly develop a medication plan prototype that is usable and support patient safety?

[Single choice: Yes/No/Do not know + Open comment box]

3a. How do you appreciate with having persons with medications, physicians and nurses working together to create a joint, patient-safe and usable medication plan prototype?

[Open question]

3b. What do you experience as difficulties for having persons with medications, physicians and nurses working together to create a joint, patient-safe and usable medication plan prototype?

[Open question]

4a. In the workshops, I was allowed to speak to the extent that I wanted.

[Grade 1-10; 1 = do not agree, 10 = totally agree // Do not know]

4b. Describe, with your own words, your possibility to speak to the extent that you wanted. We appreciate both positive and negative feedback.

[Open question]

5a. The views I expressed in the workshops were taken into account in developing the medication plan prototype

[Grade 1-10; 1 = do not agree, 10 = totally agree // Do not know]

5b. Describe, with your own words, how you felt your expressed views were managed. We appreciate both positive and negative feedback.

[Open question]

6a How did you experience the balance between how much older persons with medications, general practitioners and nurses expressed their wishes?

[Grade 1-10; 1 = very bad, 10 = excellent // Do not know + Open comment box]

6b. Describe, with your own words, how you experienced the balance between everybody’s wishes. We appreciate both positive and negative feedback.

[Open question]

7. How did you experience the balance between how much the views of older persons with medications, general practitioners and nurses were listened to?

[Grade 1-10; 1 = very bad, 10 = excellent // Do not know + Open comment box]

7b. Describe, with your own words, how your experienced the balance between how views were listened to. We appreciate both positive and negative feedback.

[Open question]

8a. How did you perceive that the information provided before, during and after the workshops facilitated your participation?

[Grade 1-10; 1 = very bad, 10 = excellent // Do not know + Open comment box]

9. How did you experience that the practical parts of the workshops (i.e. the use of the digital platform, the facilitators’ actions) facilitated your participation?

[Grade 1-10; 1 = very bad, 10 = excellent // Do not know + Open comment box]

**Final question:**

Do you have any additional comments on the prototype itself or on the co-design work that you would like to share with us?

[Open question]

Thanks for your participation in the workshops and in this survey!
