# Supplementary material for: How Older Persons and Health Care Professionals Co-designed a Medication Plan Prototype Remotely to Promote Patient Safety: Case Study
Source: JMIR Aging. 2023 Apr 7;6:e41950. doi: 10.2196/41950 (PMC10131987; doi:10.2196/41950)
Supplement: Multimedia Appendix 2 [file aging_v6i1e41950_app2.docx]

***Time-table and time-stamps from audio-recordings
Workshop 1 and 2***

**Time-table with time-stamps for workshop 1**

| **Activity** | **Time planned**  (mm:ss) | **Time performed**  (mm:ss) | **Time deviation**  (+/- mm:ss) |
| --- | --- | --- | --- |
| Introduction and presentation of participants  Familiarisation with the group and to Zoom by answering Zoom Polls about expectations of the initiative | 10:00 | 10:40 | +00:40 |
| Presentation of “Patient Contract” in general | 04.00 | 05:50 | +01:50 |
| Presentation of the aim with the initiative | 01:00 | 01:24 | +00:24 |
| Presentation of outputs, i.e. findings from the Discover phase along with information from research studies and regulations related to the initiative | 05:00 | 06:50 | +01:51 |
| **Part 1 -** Invitation to synthesise outputs into a reduced number of opportunities and requirements for a medication plan | 05:00 | 03:00 | -02:00 |
| **Part 1 -** Brainstorming in breakout rooms*. Two “user personas”, i.e. descriptions of two actual cases about older persons taking medications, were used to guide the discussion. Notes collected in Padlet | 15:00 | 15:15 | +00:15 |
| **Part 1 –** Shared debriefing from each group about desirable opportunities and requirements identified | 10:00 | 09:25 | -00:35 |
| **Part 2** - Invitation to define functions and content of a medication plan | 05:00 | 02:15 | -02:45 |
| **Part 2 -** Brainstorming in breakout rooms*. Notes collected in Padlet | 10:00 | 11:30 | +01:30 |
| **Part 2-** Shared debriefing from each group about functions and content identified | 10:00 | 11:50 | +01:50 |
| BREAK** | 10:00 | 08:00 | -02:00 |
| Brief summary presentation of part 1-2 | 05:00 | 05:10 | +00:10 |
| **Part 3a**- Clarifying Zoom Polls about opportunities and requirements | 10:00 | 08:09 | -01:51 |
| **Part 3b**- Clarifying Zoom Polls about function and content | 10:00 | 12:34 | +02:34 |
| Evaluation of the session with Zoom Polls and verbal feedback | 05:00 | 02:57 | -02:03 |
| Rounding up and information about the next step in the process | 05:00 | 03:12 | -1:48 |
| **Total time** | **2:00:00** | **1:58:02** | **-1:58** |

** Activity in three breakout rooms; healthcare professionals in two and older persons in one. All other activities were performed in the main room*

*** During the break pre-written Zoom Polls were adjusted, based on insight gathered so far, by the facilitators.*

**Time-table with time-stamps for workshop 2**

| **Activity** | **Time planned**  (h:mm:ss) | **Time performed**  (mm:ss) | **Time deviation**  (+/- mm:ss) |
| --- | --- | --- | --- |
| Introduction  Familiarisation with Padlet with a short illustration using a case where participants were invited to make their own notes in Padlet | 05:00 | 08:05 | +03:05 |
| Presentation of the design brief, informed by workshop 1, with an invitation to provide feedback by answering Zoom Polls.  Presentation of patient safety and usability | 10:00 | 08:44 | -01:16 |
| **Part 4 –** Invitation to individually apply the drafts on a fictitious case and write positive and negative feedback for each draft in Padlet | 10:00 | 10:21 | +00:21 |
| **Part 4** – Feedback loop with individual reflections collected in Padlet | 10:00 | 07:18 | -02:42 |
| **Part 4 –** Shared debriefing with feedback | 08:00 | 08:02 | +00:02 |
| BREAK | 02:00 | 00:55 | -01:05 |
| **Part 5 -** Invitation to, in a group, apply the drafts on a fictitious case and propose refinements to make them more usable and safe | 05:00 | 03:35 | -02:25 |
| **Part 5 -** Feedback loop in breakout rooms*. Notes collected in Padlet | 15:00 | 17:10 ^1^ | +02:10 |
| **Part 5 -** Shared debriefing of refinements | 10:00 | 15:11 | +05:11 |
| BREAK** | 10:00 | 09:40 | -00:20 |
| Brief summary presentation of parts 4-5 | 05:00 | 05:53 | +00:53 |
| **Part 6 –** Invitation to address potential benefits and risks on patient safety and usability of a medication plan | 05:00 | 01:47 | -03:13 |
| **Part 6** – Feedback loops with individual reflections collected in Padlet.  Shared debriefing of reflections | 10:00 | 07:38 | -02:22 |
| **Part 7 -** Clarifying Zoom Polls to prioritise requirements and content | 05:00 | 11:00 | +06:00 |
| Evaluation of the session with Zoom Polls and verbal feedback | 03:00 | 00:37 | -02:23 |
| Summarising and information about the next step in the process | 07:00 | 05:35 | -1:25 |
| **Total time** | **2:00:00** | **2:01:31** | **+1:31** |

** Activity in three breakout rooms; mixed groups with healthcare professionals and older persons All other activities were performed in the main room*

*** During the break, pre-written Zoom Polls were adjusted, based on insights gathered so far, by the facilitators.*

*1. Based on two recordings as one recording from a breakout room failed*
