# Supplementary material for: How Older Persons and Health Care Professionals Co-designed a Medication Plan Prototype Remotely to Promote Patient Safety: Case Study
Source: JMIR Aging. 2023 Apr 7;6:e41950. doi: 10.2196/41950 (PMC10131987; doi:10.2196/41950)
Supplement: Multimedia Appendix 3 [file aging_v6i1e41950_app3.docx]

**Interview-guide**

**Co-design of a medication plan**

**Aim with the interviews**

To collect participants' experiences on co-creating a medication plan prototype by asking them to reflect on the co-design work and on the outcome, i.e. the medication plan prototype.

**Introduction**

I do research on how to increase patient safety in the medication use process. As you have been involved in the co-creation of a medication plan prototype, I would like to hear your experiences of participating in this kind of work and on the outcome itself, i.e. the medication plan prototype.

The interview is intended to clarify what emerged during the co-design initiative.

**Question-guide**

The interview concerns three topics: a background question, questions about the outcome, i.e. the medication plan prototype and questions about participation in co-design work.

- **Background:**
  - What was your role in the team?
- **Questions about the medication plan prototype:**
  - What do you think was the most important outcome during this work?
  - In what way do you think a medication plan prototype could contribute to increased patient safety?
    - *In the survey, lack of time for physicians were highlighted as a possible risk. What is your thoughts about that?*
  - Do you find the result, i.e. the medication plan prototype, useful for yourself?
    If or if not – elaborate.
    - *In the survey, the availability of the medication plan has been discussed. What are your thoughts about that?*
    - *In the survey, the overview of a plan in the medication list has been discussed. What are your thoughts about that?*
- **Questions about participation in a co-design work:**
  - What does it mean for you to co-create a medication plan that should be usable and support patient safety?
  - Why did you choose to participate?
  - What is your opinion about participating in this type of co-creative work?
  - What expectations did you have on the initiative? Were they fulfilled?
    If or if not – elaborate.

**Rounding up**

Do you want to add anything that has not been addressed in these questions?
